# MEATabolomics: Muscle and Meat Metabolomics in Domestic Animals

**DOI:** 10.3390/metabo10050188

**Published:** 2020-05-11

**Authors:** Susumu Muroya, Shuji Ueda, Tomohiko Komatsu, Takuya Miyakawa, Per Ertbjerg

**Affiliations:** 1 NARO Institute of Livestock and Grassland Science, Tsukuba, Ibaraki 305-0901, Japan; 2 Graduate School of Agricultural Science, Kobe University, Hyogo 657-8501, Japan; uedas@people.kobe-u.ac.jp; 3 Livestock Research Institute of Yamagata Integrated Research Center, Shinjo, Yamagata 996-0041, Japan; komatsutom@pref.yamagata.jp; 4 Graduate School of Agricultural and Life Sciences, University of Tokyo, Bunkyo-ku, Tokyo 113-8657, Japan; atmiya@mail.ecc.u-tokyo.ac.jp; 5 Department of Food and Nutrition, University of Helsinki, 00014 Helsinki, Finland; per.ertbjerg@helsinki.fi

**Keywords:** authentication, biomarker, breed, feeding, meat quality traits, metabolite, postmortem aging, processing, skeletal muscle

## Abstract

In the past decades, metabolomics has been used to comprehensively understand a variety of food materials for improvement and assessment of food quality. Farm animal skeletal muscles and meat are one of the major targets of metabolomics for the characterization of meat and the exploration of biomarkers in the production system. For identification of potential biomarkers to control meat quality, studies of animal muscles and meat with metabolomics (MEATabolomics) has been conducted in combination with analyses of meat quality traits, focusing on specific factors associated with animal genetic background and sensory scores, or conditions in feeding system and treatments of meat in the processes such as postmortem storage, processing, and hygiene control. Currently, most of MEATabolomics approaches combine separation techniques (gas or liquid chromatography, and capillary electrophoresis)–mass spectrometry (MS) or nuclear magnetic resonance (NMR) approaches with the downstream multivariate analyses, depending on the polarity and/or hydrophobicity of the targeted metabolites. Studies employing these approaches provide useful information to monitor meat quality traits efficiently and to understand the genetic background and production system of animals behind the meat quality. MEATabolomics is expected to improve the knowledge and methodologies in animal breeding and feeding, meat storage and processing, and prediction of meat quality.

## 1. Introduction

In the last two decades, the metabolomic approach has been employed widely in the research fields of animal and plant nutrition, physiology, breeds, environment, post-harvest storage and processing [1,2] with the advantages of its high-throughput capacity. The target molecules of food metabolomics are animal and plant metabolites, including low molecular-weight hydrophilic and hydrophobic compounds. These compounds include key metabolites such as flavor-associated compounds, nutrients, and functionality-associated compounds in food, some of which have a molecular weight of >1000. Such compounds characterize nutritional and sensory properties, and therefore the metabolome of a food provides important phenotypic information. In this context, metabolomics is a powerful tool to obtain a deeper understanding of the biologically and agriculturally meaningful information in the global metabolome profiles and the changes caused by factors in food production processes [3].

Currently, two major types of platforms have been applied for metabolomic studies so far: mass spectrometry (MS)-based [4] and non-MS-based techniques such as nuclear magnetic resonance (NMR) [2]. Moreover, various types of separation techniques are incorporated in most of the MS-based approaches, depending on the lipophilicity and polarity of the metabolites of interest. Combined with advanced statistical analyses, multivariate analyses, and bioinformatic databases, metabolomics provides clues not only to discover biomarkers for monitoring and assessment of food quality but also to uncover molecular pathways in which enzymatic reaction generates key metabolites [5]. Correlation analysis between sensory evaluation scores and metabolomic profiles potentially leads to key compounds that are associated with eating quality, such as flavor and texture [6,7,8], which may enable us to predict the palatability of the foods by using the biomarker metabolites in pre- and post-harvest materials. Metabolomic profile data have also been utilized to explore the responsible genes for specific metabolite-featured phenotypes in genome-wide association studies (GWAS) [9,10].

In recent years, studies in muscle and meat science have utilized metabolomics as in other fields [11,12,13]. Skeletal muscle characteristics are designed by a functionally cooperative set of genes specific to the spatiotemporal requirement in each muscle. The gene expression is further modulated at levels of transcription, post-transcription, translation, and protein modification during development, growth, and maturation stages of the muscle. Accordingly, muscle metabolites determine the physiological muscle characteristics and meat quality traits as the major phenotypic components. The history of the muscle in the developmental and physiological specialization, feeding processes of animals, circumstances in slaughtering, postmortem aging, and processing processes all have influences on transcriptomic and/or proteomic profiles, and finally on the muscle metabolome profile. This makes the understanding of mechanisms behind meat quality traits by the use of molecular markers more difficult. Nevertheless, the metabolome profiles have been successfully distinguished with metabolites of biomarker candidates in comparisons such as between physiologically different types of skeletal muscles [14,15,16], between at-slaughter and postmortem muscles [14,15,17,18,19], between meats produced by different animal feeding conditions [20,21,22,23,24,25,26], and between different types of meat processing [7,27,28,29,30]. Thus, to better understand factors determining muscle characteristics and meat quality, the recent metabolomics approach has great advantages, which potentially supports to capture biomarkers of the phenotype. Since raw and cooked meat are rich in flavor-associated volatile compounds and precursors, MEATabolomics studies in combination with sensory evaluation have been conducted to explore biomarker candidates associated with eating quality of meat.

Especially in studies of postmortem muscle and meat quality, MEATabolomics is expected to provide a clue for drawing maps of the metabolic network in postmortem muscle aging and flavor development during cooking, which cannot be replaced by the other biological or chemical approaches. Muscles, especially beef and pork, are more or less aged after death for the tenderization and flavor component accumulation. During the aging, the muscles experience drastic and irreversible physico-chemical and metabolic changes [31]. These metabolic changes have a large impact on subsequent beef and pork quality, under influences of physiological background before slaughter. Despite the impact on meat quality, the metabolic changes have not easily predicted, because coordinated metabolism in live muscle is no longer maintained due to lack of energy supply and arrest of de novo gene expression after animal death. Postmortem metabolic maps drawn by MEATabolomics could lead to a finding of the metabolic factors responsible for meat quality traits and thereby contribute to the exploration of biomarkers in quality monitoring, processing, and authentication of meat including chicken. Thus, postmortem muscle metabolism and the factors associated with meat quality have been the major and unique challenges to be addressed for MEATabolomics.

This review provides an overview of past applications, recent findings, and topics achieved by MS- and NMR-based MEATabolomics approaches associated with animal muscle physiology and meat quality traits. We especially focus on studies of meat quality traits and the factors influencing meat quality, such as postmortem aging, as well as animal breed and feeding, processing, spoilage, and authentication. Using those keywords, a total of 78 studies were collected by search in databases such as PubMed, ScienceDirect, and Springer Link, from which 56 studies were selected and featured.

## 2. MEATabolomic Methodologies and Approaches

The MS-based metabolomics need a separation step suitable for target metabolites of interest (Table 1). Of the current separation techniques, capillary electrophoresis (CE)–MS has high performance in the acquisition of polar and charged metabolite information with high resolution and sensitivity, but not in non-ionic molecules [32,33]. CE has an advantage over gas chromatography (GC) and high-performance liquid chromatography (HPLC; LC) for the resolution of ionic compounds, including their isomers, owing to separation in CE by their charge-to-mass ratio. GC–MS has been widely used for decades due to its established high-separation efficiency, selective and sensitive mass detection, and a broad range of target molecules, mainly fatty acids and sugars, although it needs derivatization of sample molecules, especially for non-volatile compounds. Derivatization artifacts may be generated by decomposition of thermolabile molecules in GC, during the analysis after enhancement of volatility of molecules. On the other hand, LC–MS targets compounds with a relatively narrow range but has the flexibility to change the type of targets with replaceable separation columns. As the common stationary columns in LC, C18 reversed-phase is the most frequently used for separation of hydrophobic molecules, while the polar phase, such as silica and amide, is used for hydrophilic molecules. Running time of LC separation can be much reduced to less than 10 min in the case of ultra HPLC (UHPLC, UPLC).

The separated molecules are required to be ionized generally by electrospray ionization (ESI) in CE, electron ionization (EI) or chemical ionization (CI) in GC, and ESI or atmospheric pressure chemical ionization (APCI; API) in LC. MS detection techniques have been developed to various types, and, currently, the most frequently used technique for matching to the upstream CE, GC, and LC separation steps is time-of-flight (TOF) to achieve higher sensitivity, accuracy, rate of measurement, and mass and dynamic range for the acquisition of more metabolite information [4]. Fourier transform (FT) type MS also has a high-throughput performance with high resolution and a broad range of target molecules and is thereby used for metabolomics. Of the FTMS, FT-ion cyclotron resonance (FT-ICR) MS utilizes a magnetic field to detect resonance of cyclotron motion of metabolite ion, while the orbitrap type is based on a system using an electric field. In some cases, tandem MS (MS/MS) is employed to acquire more structural information for the characterization of compounds. These MS-based approaches are utilized not only for targeted metabolomics but also for untargeted metabolomics [26,34,35]. Recently, rapid evaporative ionization mass spectrometry (REIMS) has been introduced in MEATabolomics [26,36]. This method allows direct MS analysis of a biological sample with no preparative steps, under normal atmospheric conditions, due to being based on the ambient ionization [37,38]. A trend of MEATabolomic approach can be seen in the times of employment for each technique in the 56 studies collected in this review: CE–MS, GC–MS, LC–MS, NMR, and REIMS approaches have been employed in 5, 17, 17, 22, and 1 studies, respectively, with some cases combining multiple approaches (Table 2).

After MS measurement, initial putative metabolite can be identified on the basis of the accurate mass–to–charge ratio (m/z) of the mass spectral ion in MS-based metabolomics. This is assisted by the use of metabolite databases such as METLIN (https://metlin.scripps.edu/), the Human Metabolome Database (HMDB; http://www.hmdb.ca/), and MassBank (http://www.massbank.jp/).

NMR is also highlighted as a method for practical use, such as authentication purposes, in an analytical routine. This technique can provide rapid and reproducible measurements in complex mixtures without a time-consuming pretreatment. Although NMR has relatively low ability to profile metabolites compared to the MS-based techniques due to its low resolution and sensitivity, it has the ability to collect distinct information that the other metabolomics cannot access in a non-destructive and non-biased way [2]. Especially, approaches with ^1^H–high-resolution magic angle spinning (HR–MAS) have also been applied to characterize meat quality [39,46,59]. Unlike the other types of NMR, this NMR enables us to investigate intact tissue specimens (10–50 mg) and allows the spectra to be obtained with a high resolution compatible to that obtained in liquid samples in less than 30 min.

Once the data matrix is produced from the collected raw data, subsequent statistical analyses and data mining are often performed to identify samples or variables (metabolites) that characterize the variations between datasets and may represent biologically meaningful determinants. In most cases, the statistical sample classification is conducted to overview pattern recognition of sample categories, by multivariate analyses such as principal component analysis (PCA), clustering analysis, partial least square analysis (also called projection to latent structures, PLS), PLS–discriminant analysis (PLS–DA), and random forests (RF) [5,75].

PCA is an unsupervised statistical method that reduces dimensions of high dimensional data to visualize sample distribution and grouping on the principal component (PC) plot based on the pattern of the metabolite dataset and thereby is employed by most of the metabolomic studies as the first step of data analysis [75]. Clustering analysis, especially hierarchical clustering analysis (HCA), is also widely used to generate a snapshot profiling of dataset. In HCA, the algorithm divides the measured datasets into subgroups so that datasets with similar metabolomic profiles are placed in each group. PCA and HCA are frequently used for visualization of classification in omics studies, including metabolomics.

PLS has been developed as a supervised extension of PCA [75]. This regression-based method is especially useful when fewer samples are available than measured metabolites. PLS–DA is used to elucidate the metabolites that carry the information of classification, which screens highly contributing metabolites to the classification. PLS and PLS–DA have often been employed to sharpen the separation between the groups, especially when the groups are not sufficiently separated in PCA. Orthogonal PLS (OPLS)–DA is an extension of PLS–DA to cover the defect of PLS–DA, owing to its robustness against noise. RF is a machine learning method used to discriminate two groups. Different from conventional methods such as PCA and PLS–DA, RF allows data structure understood without dimensional reduction with its low bias and low variance. As with other data mining analyses, the application of support vector machine (SVM) and neural networks are attempted for some of MEATabolomic studies [34]. These analytical methods have been applied for sample characterization and determination of biomarker candidates.

Classical statistical analyses such as Student’s *t*-test and analysis of variance (ANOVA) are also used to compare metabolite levels between sample groups, with care about the detection of false-positive metabolites in multiple comparisons of the datasets. To reduce false-positive detection caused by the familywise error rate (FWER) in multiple comparisons, procedures of false discovery rate (FDR), Holm, Bonferroni, or Benjamini–Hochberg are applied to adjust the levels of significance detection in metabolomic studies [5]. Obviously, there are advantages and disadvantages to every method and database. Further information and details in these statistical analyses are described for reference in methodological reviews [5,75].

Moreover, recent progress of bioinformatics analytic tools and databases largely contributes to compound annotation, metabolic pathway finding, and biological data interpretation of the extracted compounds (see the websites of The Metabolomics Society, Inc. for more detail information; http://metabolomicssociety.org/). HMDB (http://www.hmdb.ca/) and PubChem (https://pubchem.ncbi.nlm.nih.gov/) are among the most widely used databases, and they play important roles in the annotation of compound signals.

The Kyoto Encyclopedia of Genes and Genomes (KEGG; https://www.genome.jp/kegg/) is frequently used to connect the metabolome profile with genomic, transcriptomic, enzymatic, and pathway information, which supports prediction and pathway mapping of potential molecular factors responsible for the phenotypic events. Software for analyses of metabolic pathways such as MetaboAnalyst (https://www.metaboanalyst.ca/) has also often been used for biological interpretation in recent years. MetaboAnalyst provides a variety of statistical analysis tools, including visualizing programs, multivariate analyses, and metabolite pathway analyses. These metabolomic approaches, especially CE–MS, GC–MS, LC–MS, and NMR, in combination with subsequent data analyses have recently been increasingly used in animal-based food research fields [10,11,12,76].

## 3. Metabolomes Associated with Meat Quality Traits

Metabolomics is used for the exploration of key compounds contributing to the physico-chemical properties and sensory evaluation scores, and thereby it contributes to accounting for meat palatability and quality traits such as color and water-holding capacity (WHC). As described below, the contents of skeletal muscle metabolites, including amino acids and sugars, are affected by animal genetic background, feeding, muscle type, postmortem aging, and meat processing. These metabolomic changes are linked with the physico-chemical properties of muscles and meat, as shown in variations in meat color and WHC between different types of muscles or between differently treated muscles. Accordingly, the metabolites are beneficial indicators to predict the physico-chemical meat quality traits, which indicate a result more or less from the influences of animal genetic background, feeding, and postmortem processes (Table 2).

### 3.1. Meat Color, WHC, and pH Decline

The color of meat is one of the commercially important meat quality traits since the appearance of meat has a great influence on the consumer’s desire to purchase [77]. The redness and discoloration are determined by the chemical status of myoglobin, which is affected by multiple factors that are inherent to both live animal and postmortem conditions. With hydrophilic interaction liquid chromatography–mass spectrometry (HILIC–MS)-based metabolomics, influences of postmortem aging time, packaging atmosphere, and display time on lamb meat color was investigated to identify the metabolites affected by those postmortem conditions [40]. Many compounds were found to change with time of aging and display or packaging atmosphere, which indicated the contribution of the compounds, including amino acids, sugars, nucleotides, and organic acids, to the sample discrimination. Furthermore, the abundance of reducing or antioxidant compounds such as L-glutathione and taurine contributed to color stability. Intriguingly, boron complexes of sugar and malate, presumably color stability-related compounds present in plants but not in animals, were detected, although the role of the compounds remains unknown [40]. WHC is also one of the most important traits of meat quality. Various genetic and environmental factors can induce myofibrillar protein denaturation and thereby lower the WHC of meat. Meat with lower WHC generally has higher drip loss, which has a negative impact on juiciness and palatability of the meat. To assess the responsible genes for WHC in pork, metabolomics was applied to characterize pork with high drip loss via metabolic pathway enrichment analysis in an integrative omics approach, as described below (see Section 4.1) [35].

Due to the large impact on meat color, WHC, and other final meat quality traits, postmortem pH decline is also an important factor to be controlled. In a study investigating two chicken lines differing in ultimate pH (pHu), the *pectoralis major* muscles of the two lines were discriminated against by high-resolution NMR metabolomics [41]. Subsequent metabolite set enrichment analysis (MSEA) of the data showed that carbohydrate metabolism in the low pHu line and metabolism of amino acid and protein in the high pHu line were over-representative pathways. The difference in the metabolome profile between high and low pHu chicken might be due to the ability of the muscles of glycogen storage and carbohydrate use.

### 3.2. Flavor and Palatability

Skeletal muscle metabolites include amino acids and sugars that are precursors of volatile compounds associated with meat aroma. The contents of these compounds are altered, depending on animal feeding, the genetic background, and the postmortem aging process. This could cause variations in meat flavor between meats from different animal production or postmortem conditions. Therefore, muscle and meat metabolites are useful indices to predict or evaluate meat flavor and overall palatability in a comparison between meats of different animal breeds, feeding conditions, and/or postmortem processes. After postmortem aging, amino acids and sugars in muscles make a large contribution to the quality of cooked meat, both as intact forms and as products by the Maillard reaction that occurs between amino compounds and reducing sugars during heat treatment. One of the final volatile products, 2,5-dimethyl-4-hydroxy-3(2H)-furanone (DMHF; furaneol), has been identified as a key aroma compound in beef extract by aroma extract dilution analysis (AEDA) [42]. Even though the aroma of furaneol depends on its concentration, it has great influences on sensory characteristics as a flavor component in meat as well as other foods [78]. In fact, furaneol was detected on Japanese Shorthorn beef during cooking at 180 °C by analysis with headspace solid-phase micro-extraction (HS/SPME) GC–MS, as well as other flavor-associated volatile compounds [43]. Moreover, inosine-5′-monophosphate (5′-IMP) greatly contributes to the enhanced flavor of the meat due to its primary role as an *umami* compound, in a different manner from aroma compounds [79,80]. Thus, the MEATabolomics approach provides tools to access these palatability-associated compounds with the high-throughput analytic systems.

In a beef study combined with flavor-associated evaluation scores, the *longissimus thoracis* (LT) muscles of Japanese Black (JB) and Holstein cattle were discriminated by the metabolome profiles especially with decanoic acid and glutamine [44], indicating that these compounds were associated with the difference in flavor between the two breeds. Metabolites in beef affecting the sensory attributes have also been explored using commercial ground beef differing in grinding and packaging methods [60]. The study resulted in the determination of 33 metabolites differentiating the grinding and 22 metabolites associated with beef flavor and off-flavor. Results from a pork study of differences between breeds indicated that high carnosine content in meat was associated with a low flavor/taste score in pork [6]. Sensory score-associated variation of sugar content and composition of fatty acids, amino acids, and nucleotides were also observed in a comprehensive analysis of the metabolites in seven brands of JB beef [61]. The results of these studies suggest the influences of animal genetic and feeding factors on sensory evaluation scores via the meat metabolites. It is quite likely that altered contents of amino acids and sugars in the animal and meat-producing processes cause changes in the content of volatile flavor-associated compounds, including furaneol in the cooked meat. Even in the presence of complicated biological and manufactural factors, these studies revealed that metabolomic information is expected to provide indices to predict sensory phenotypes of meat.

### 3.3. Chicken Meat Quality Traits

Chicken meat quality traits are one of the most intensively focused targets in recent MEATabolomics, due to the globally growing market. A Chinese local breed, Wuding chicken meat, was analyzed by the ^1^H NMR-based approach [45], in which the age of chicken affected the levels of metabolites such as lactate, creatine, IMP, glucose, carnosine, anserine, and taurine. Moreover, the abnormal meat quality of chicken is currently an important issue in the poultry industry. Increasing incidents of muscle abnormalities, including white striping (WS), wooden breast (WB), and spaghetti meat (SM) phenotypes, have been linked to a drastic improvement of breast muscle mass [81]. Metabolomics approaches have been applied to especially elucidate the mechanisms underlying the hardness of the WB phenotype. As WB-related biomarker candidates, Abasht et al. [47] identified compounds associated with protein levels, muscle protein degradation, and altered glucose metabolism, using GC–MS and LC–MS/MS followed by RF procedure analysis. Another study with ^1^H–NMR approach revealed that WB-affected broiler chicken breasts had a higher content of leucine, valine, alanine, glutamine, lysine, lactate, succinate, taurine, glucose, and IMP, but lower histidine, β-alanine, acetate, creatine, creatinine, and anserine compared to normal fillets [48]. In another attempt to explore WB myopathy biomarkers, muscle exudate was used as a sample. Linked with discrimination of the samples between WB and non-WB phenotypes, amino acids, nucleotides, and organic acids were identified as the WB-associated metabolites by an NMR approach [49], suggesting the usefulness of those metabolites as the WB markers. This result was partly consistent with the previous study using breast samples with/without WB [48]. WS, on the other hand, has been associated with altered metabolism related to carbohydrates, long-chain fatty acids, and carnitine in a study with GC–MS and LC–MS approaches, suggesting the involvement of altered β-oxidation in the WS phenotype [50]. A HR–MAS NMR approach revealed that muscle dystrophy of pectoralis in chicken is associated with low content of anserine and carnosine [46]. Thus, the results of these MEATabolomics studies have suggested that the chicken breast muscle abnormalities could be caused by the altered metabolism of carbohydrates, protein and amino acids, and β-oxidation.

## 4. Factors Affecting Muscle and Meat Metabolomes

As mentioned above, meat is originally skeletal muscles of livestock; consequently, meat quality is greatly affected by factors in the livestock production system, such as animal genetic background, the feeding and stress that animals experienced, but also the physiological characteristics of live muscle, postmortem aging, processing, and spoilage of meat. All the influences of these factors can be assessed by the muscle or meat metabolome profile that is the phenotype resulting from animal experiences through the expression of gene transcription, translation, and the protein modifications as well.

### 4.1. Animal Species, Breeds, and Genetic Backgrounds

A variety of characteristics in meat depends on species or breeds of animals to a great extent. The genetic background of animals thus has an influence on the phenotype expression resulting in an inherent metabolome profile. For the purpose of investigating the effect of genetic background, metabolomics has so far been applied to capture muscle and meat phenotypic differences between species (beef, pork, chicken) [44] or breeds in beef cattle [44] and pigs [6]. The meat of those species was discriminated by PCA of GC–MS metabolome profiles with specific compounds being characteristic to each species [44], although different postmortem muscle metabolism might affect the profile. In comparison between Holstein and the highly marbled JB cattle that differ in oily flavor, sweet flavor, wagyu beef aroma, and the overall evaluation in a sensory test, their LT muscles were discriminated by OPLS–DA [44]. A higher level of decanoic acid in JB than in Holstein cattle, and differences between skeletal muscle, inter-, and intramuscular fat tissues of JB cattle were also observed.

Regarding cattle, in the comparison between different strains of Nellore cattle genetically selected by precocity, a genetic effect on *longissimus lumborum* (LL) muscle metabolome was observed by ^1^H–NMR [51]. The purpose of that study was to investigate changes in metabolites associated with muscle physiology and quality of the LL meat, under the established improving effect of high growth or precocity background on daily gain, carcass characteristics, and other industrial meaningful traits. In the subsequent PLS–DA using the detected compounds (metabolites related to glycolysis and the citric acid cycle, amino acids, organic acids, nucleotides, and sugars), the muscles were discriminated between high and low precocity groups. Pathway analysis in the study highlighted the association of the muscle protein metabolism with the intensity of the selection. Thus, these MEATabolomic studies have revealed their potential to uncover species- or breed-specific differences in metabolites, through which differences in meat quality between breeds can be assessed at the molecular level.

A study comparing ^1^H–NMR metabolome and sensory evaluation scores between five crossbreds of pigs observed an inter-crossbreed effect on metabolites, such as amino acids, lactate, IMP, glycerol, and choline-containing compounds. Some of those compounds were associated with meat quality, including sensory scores [6]. The results also suggested the association of live muscle metabolism, membrane properties, muscle fiber glycolytic potential, lipolysis, and proteolysis with the metabolomic difference.

Furthermore, in a study on pork investigating the relationship between metabolites and drip loss, metabolomics was applied in an integrative way to understand the association of a high drip loss phenotype with the genetic background [35]. In this single nucleotide polymorphism (SNP)-based genome-wide association study (GWAS), a region of candidate genes was identified on chromosome 18 as one associated with drip loss, and the metabolite glycine. Compared to conventional genetic studies using quantitative trait loci (QTL) and candidate genes for drip loss in pigs, GWAS is expected to improve the efficiency of candidate gene detection and accuracy of genomic prediction by avoiding detection of false-positive associations. In beef, GWAS was conducted on JB cattle to explore genes responsible for the palatability of beef, which revealed that SNPs in the ecto-5′-nucleotidase (NT5E) gene affected the content of IMP in the postmortem aged beef, due to the modulating effects of NT5E enzymatic activity. This is a result of which GC- and LC-based metabolomics contributed to GWAS on meat [52,53]. In these comprehensive omics approaches such as GWAS, metabolomics is useful to capture phenotypic metabolites for the candidate genes, due to its wide range of phenotypic molecular measurements.

### 4.2. Animal Feeding

Feeding conditions, one of the environmental factors for animals, have great influences on animal physiology, including skeletal muscle growth and maturation, and the final meat yield and quality. As the feeding factors, the nutritional condition and stress originating from the farming system are considered as the most important ones [82,83], due to their influences on downstream meat production. The influence of feeding on animals are expected to be observed as metabolome changes via gene expression and physiological alteration in tissues. For these reasons, metabolomics approaches have attempted to capture metabolite signatures of feeding systems aiming at the improvement of meat production.

In beef cattle, to address an increasing demand by consumers for beef produced in a sustainable farming system, the effect of differences in feeding on the muscle metabolome was assessed by metabolomic approaches with GC–MS and LC–MS/MS [20]. Between grass- and grain-fed Angus steers, significant variations between the cattle groups was observed in a variety of lipids, including polyunsaturated fatty acid (PUFA) in the *latissimus dorsi* muscle, showing higher ω3 and lower ω6 fatty acids in grass-fed cattle than in the grain-fed. The PCA classified these samples by the diet, and RF analysis based on the metabolites resulted in a predictive accuracy of 100% between the dietary conditions, in which lipids and amino acids were potential biomarkers discriminating the two groups of cattle. Intriguingly, the cortisol level was lower in grass-fed cattle, suggesting they might experience less stress than the grain-fed [20]. These results indicated that metabolite signatures could be utilized as indices not only in feeding-originated beef quality but also in animal welfare and authentication. In other studies, the effects of dietary amino acids and mate extract administration on beef were also investigated by NMR approaches followed by PCA [21,22]. Biopsy *semitendinosus* muscle samples of dairy calves fed protein-based milk replacer at 7 weeks of age were classified by amino acid supplementation [21]. Additionally, in a study of feedlot Nellore cattle, the dietary addition of mate extract, one of the antioxidant additives administered to broiler chickens, resulted in increased content of IMP, creatine, carnosine, and conjugated linoleic acid in the LT muscle, and some of these were in a dose-dependent manner [22]. Coupled with redox status analysis, the results further indicated that the beef of mate extract had increased oxidative stability, tenderness, and consumer acceptability. Thus, not only the global feeding conditions but also nutrients and ingredients in the animal feed cause changes in muscle and meat metabolomes, which can be assessed by MEATabolomics. In such studies, metabolites can be screened and be identified as potential biomarkers of the feeding.

Regarding pork, a GC–MS approach was applied to the screening of pigs fed with Clenbuterol, a β-adrenergic agonist [23]. The *biceps femoris* muscles of pigs treated with Clenbuterol were discriminated from the counterparts in the subsequent PLS-DA by compounds associated with fatty acids and amino acids. Recently, a similar study was conducted using a REIMS approach [26], in which carcasses of pigs fed with ractopamine, another β-agonist, were discriminated by the lipid profiles of REIMS with high accuracy of classification in three different types of muscle. Due to the high-throughput and accurate performance, this methodology can be widely applied to fields of practical meat research.

Such nutrimetabolomic approaches have also been attempted in chicken, of which growth performance, breast yield, and meat quality are improved with dietary lysine supplementation [12]. Aiming at an increase in flavor compounds in chicken, the effect of dietary lysine supplementation at a 1.5-fold level of a standard requirement on the breast muscle was investigated by a CE–MS approach. Supplementation resulted in increased levels of lysine degradation products, such as saccharopine, α-amino adipic acid, and glutamate, the latter being one of the most important taste-improving amino acids in meat [24]. On the other hand, in a study investigating the effect of duck aging on the meat metabolome, duck meat samples of different ages were classified in PCA and PLS–DA by NMR metabolite data, showing increased levels of lactate and anserine and decreased levels of fumarate, betaine, taurine, and inosine with increasing age [25].

### 4.3. Muscle Type

The type of skeletal muscle of pig and cattle is associated with meat quality (WHC, meat color, and tenderness) [84,85] and sensory traits [86,87]. This is due to the differences in physiological characteristics [88,89] and compositions of proteins [90,91] and metabolites [14,54,92,93] between different types of muscles that have distinct compositions of fast and slow type muscle fibers. Due to the different physiological properties of the muscle fibers, muscles with different fast/slow fiber type composition undergo different postmortem aging processes, as shown in protein [94,95] and metabolite [14,16] degradation, which could be the cause of the intermuscular difference in meat quality. Contents of flavor-associated metabolites such as amino acids, sugar, and nucleotides differed between porcine LL and *vastus intermedius* (VI) muscles in a CE–MS metabolomics study [14], and between bovine muscles in an LC–MS [15] or a UPLC–MS/MS study [16]. These results indicate the association of fast and slow type fiber composition with contents of flavor-associated metabolites in aged meat [14,15]. It is noticeable that the rate and extent of the postmortem metabolite changes including glycolysis, metabolism of amino acids and nucleotides are different between the muscle types, as shown in different accumulation of lactate in glycolysis, hypoxanthine in purine metabolism, and aliphatic amino acids and methionine in amino acid metabolism [14]. During postmortem aging of pork, thiamine is accumulated, while thiamine triphosphate level declines, which is another difference between muscle types [54]. In addition, the metabolomic difference between *longissimus* and *semitendinosus* muscles of four cattle breeds was investigated by an HR–MAS NMR approach, which resulted in a good classification of the two muscles in Chianina and Buffalo, but not in Holstein Friesian and Maremmana cattle [39]. Such intermuscular differences may be associated with differences in meat quality and sensory traits between those muscles.

### 4.4. Postmortem Aging and Storage

Aging and storage of postmortem muscle have great influences on meat quality, as well as animal genetic background and feeding conditions. Even when an animal has muscles of high meat quality potential, the final meat quality could be lowered by inappropriate management, resulting in meat deterioration such as spoilage and discoloration in parallel with abnormal metabolome changes. Inversely, the development of metabolites in meat during postmortem aging under the appropriate condition has a beneficial influence on the final meat quality.

**Figure 1:**
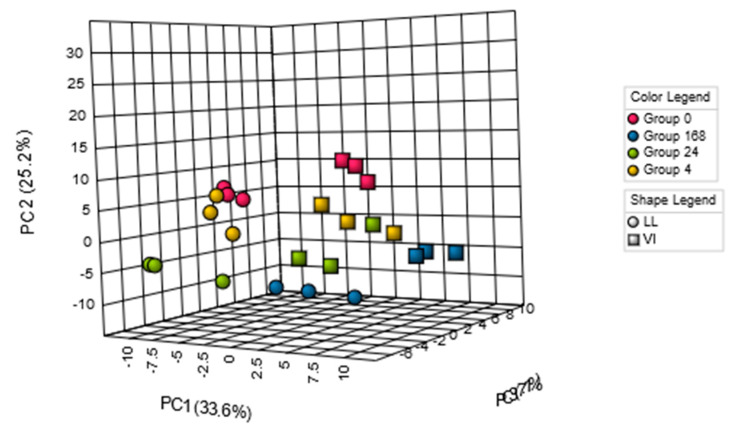
Classification of pig LL and VI muscle samples of different postmortem aging period by PCA. Pig LL and VI muscles were aged during 0, 4, 24, 168 h postmortem.

**Figure 2:**
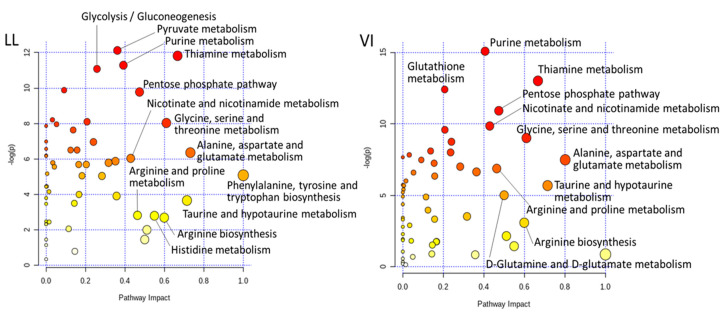
Metabolic pathway impact analysis of pig LL and VI muscles during postmortem aging. Pathway impact values (horizontal axis) were calculated from the pathway topology analysis in MetaboAnalyst. A pathway with small *p* value and high impact is plotted as a red and large circle.

MEATabolomics has contributed to postmortem meat aging studies to account for influences of postmortem aging on meat quality in terms of metabolites. In pork, postmortem changes in the LL and VI muscles were investigated by a CE–MS approach [14]. The result of this study indicated that a variety of postmortem muscle metabolisms were activated, especially within 24 h after slaughter. The PCA classified the samples primarily into LL and VI muscles, and secondarily to at-slaughter and day 14 (168 h) aging points (Figure 1). In the LL samples, intermediate glycolytic products, glucose 6-phosphate and fructose 6-phosphate (F-6P), increased until 24 h postmortem, while the downstream compounds such as fructose 1,6-bisphosphate (F-1,6P) and phosphoenolpyruvate decreased with being almost exhausted at 24 h postmortem, indicating the rate-determining activity of phosphofructokinase (F-6P → F-1,6P) in postmortem LL muscle glycolysis. Additionally, in the LL muscle, most of the amino acids and identified dipeptides increased after day 1 during the aging, suggesting that protein degradation began to be dominant after day 1. Regarding ATP degradation, marked differences in compound levels were observed in the pathways of ADP → AMP → IMP → inosine → hypoxanthine between the LL and VI muscles, suggesting the intermuscular differences in enzymatic activities in the pathways [14]. These pathways in pig LL and VI muscles were overrepresented in the analysis of pathway impact by MetaboAnalyst (Figure 2), indicating a number of biologically meaningful differences in metabolic pathways between the two muscles. Such pathway analyses further afforded new insights: pathways associated with thiamine, pentose phosphate, and NADH metabolism had a high impact on postmortem aging in both muscles. In other studies analyzing bovine LL, *psoas major* (PM), and/or *semimembranosus* muscles, intermuscular metabolomic differences in energy metabolism during 24 h aging [16], and differences in metabolites associated with meat color and lipid oxidation during 23 d aging [15] were also observed by LC–MS-based approaches. Thus, the skeletal muscle type is associated with the muscle metabolome as described above, in which postmortem aging makes the intermuscular metabolome differences inherent to live animals further complicated, as shown in the complexity of the differences in the final meat quality. Nevertheless, MEATabolomics approaches have shown its advantages to discriminate meat samples by muscle type and postmortem aging conditions.

Regarding beef, metabolomic changes during postmortem aging was also analyzed by CE–MS [17] and NMR approaches [18,19] in *longissimus* muscle. In the CE–MS metabolomics targeting charged metabolites, changes in nutritionally important compounds such as choline, nicotinamide, and thiamine were observed during 14 d aging of JB cattle beef, some of which contributed to classification of the samples by the aging period [17]. On the other hand, NMR studies were conducted for metabolomic profiling during 10 weeks [18] and 21 days [19] postmortem. The former NMR study aimed to explore excellent biomarkers of postmortem JB beef aged up to 10 weeks, and revealed that leucine and creatine are available as biomarkers for long-term aging (>2 weeks) because of positive and negative correlation with aging duration, respectively [18]. The L-value, defined as the ratio of leucine to creatine in the study, was more appropriate to evaluate meat quality during long-term aging than the conventional K-value that is calculated by using the contents of ATP, ADP, AMP, and IMP. In addition, this study also showed that the NMR metabolomics was able to estimate unsaturation degree of triacylglycerol that was correlated to content ratio of unsaturated fatty acids (R^2^ = 0.944) and melting point of intramuscular fat (R^2^ = 0.871). This suggests that such an approach can evaluate not only postmortem meat aging and intramuscular fat content, but also palatability-associated quality traits such as juiciness and WHC that might be assessed through NMR-measured pH profile [18] in highly marbled JB beef. Besides, biomarker candidates for prolonged postmortem aging (up to 44 days) of Piedmontese LT beef was explored by the LC–MS approach [55]. The results indicated the possibility of glutamate, serine, and arginine being candidates of postmortem aging indicators due to their constant increase until day 44. The prolonged aging period likely enhanced the accumulation of amino acids and some other metabolites. In these studies, metabolomics approaches combined with statistical analyses have enabled screening of metabolic biomarker candidates of postmortem aging from the meat metabolome dataset. Thus, metabolomics have contributed not only to the understanding of postmortem muscle metabolisms but also to the finding of better biomarkers that the conventional studies have never assessed. These MEATabolomics of postmortem aging of pork and beef re-emphasize the possibility that the amino acid generation during cold storage is primarily due to postmortem protein degradation in the muscles, as shown in previous studies of aging [31,94,95,96,97,98,99,100,101]. Besides, the elucidation of flavor-associated compound generation from muscle metabolites has been accelerated with the accumulating knowledge of the uncovered meat metabolomes through postmortem aging studies on cooked meat [43].

Other optional postmortem storage conditions are also to be investigated due to the influences on a variety of metabolites associated with meat quality traits. A dry-aging condition of 3 °C with 0.2 m/s air-velocity for 3 weeks showed high improvement of beef palatability in a sensory panel evaluation compared to conditions of general wet-aging and other dry conditions, with higher contents of compounds including branched amino acids and IMP in a study by an NMR approach [56]. The air flow of this storage method might simply accelerate the up-concentration of the flavor-associated metabolites, but other undetermined factors are likely also involved. On the other hand, the chilling rate of lamb LT muscle during aging affected the muscle energy metabolism within 24 h postmortem, which was associated with meat tenderness in a study utilizing ^1^H–, ^31^P–NMR, and LC–MS [57]. MEATabolomics was also conducted to investigate roles of NADH in discoloration mechanism via mitochondria activity during postmortem wet-aging of bovine LL muscle [58]. The results suggested that discoloration during postmortem aging of beef was due to increased oxidative stress, mitochondrial damages, and decreasing metabolite sources to regenerate NADH.

### 4.5. Processing

Meat processing is important to improve the microbiological safety, color, flavor, and texture for the development of favorable meat products. Product chemical information in processing is useful to monitor the processing steps for quality management and to design better products for quality development. To date, metabolomics has been used for the characterization of processed products in studies that assess effects of marinating conditions of chicken breast fillets [7,29] and the marination time on pork [30], and processing conditions on dry-cured hams [27,28] and fermented sausages [59], and texture defects in dry-cured ham [102]. Metabolomic profiling of hams from Japan and European countries identified a total of 203 charged metabolites by CE–MS, and revealed that redness and fat whiteness are associated with metabolomic profiles. The result of this study suggested that the metabolome of hams might be affected by country of origin and processing methods such as smoking and use of starter culture [27]. Difference between Chinese dry-cured and other hams were investigated by NMR metabolomics, in which a total of 33 charged metabolites were identified and each ham was characterized by a specific metabolite set [28]. In an application to a traditional Spanish fermented *salchichón* sausage, a HR–MAS NMR approach showed that the metabolome profile changed, depending primarily on the fermentation and secondarily on the drying process [59]. This result suggested that proteolysis and lipolysis attributed to microbial activities could be monitored by NMR metabolomics.

Metabolomics was also used to test the effect of marination and storage conditions on preservation and sensory quality of chicken breast fillets. By comparison between different types of marinade, temperatures, and intervals during marinating process, each marinade was characterized by a distinct organic acid profile [29]. The results of this study also showed that marinating time has influence on the indigenous microorganisms and the sensory characteristics. A subsequent study to test the effect of the processing conditions (marinade type, storage, and microbial load) showed that the profiles of organic acid and volatile compounds were discriminated between pomegranate-based marinated and control samples according to storage time, microbial load, and sensory score [7]. On the other hand, in a Chinese traditional marinated meat product, amino acids, sugars, acetate, succinate, uracil and inosine increased during marinating, while lactate, creatine, IMP and anserine decreased [30]. In this study, combined with sensory test, a negative effect on the taste of marinating meat in soy sauce was observed during the late stage of dry-ripening, accompanying decreases in most of the metabolites, which suggests that shortening the dry-ripening period could be better to improve the taste quality.

### 4.6. Spoilage

Spoilage developed by microbiological activity has been the greatest concern for consumers in terms of meat safety. Meat spoilage is developed through microbial growth during meat cutting, storage, and distribution processes after the slaughter of animals. Microbial growth causes chemical changes that result from changes in the microorganism itself and the metabolic output on meat. Therefore, chemical information acquired by metabolomics is expected to be utilized to quantify the degree of spoilage and predict the number or activity of microorganisms [103]. As a candidate of such compound set, volatile organic compounds (VOC) have been focused on in metabolomic studies of spoilage. In an attempt targeting microorganism-associated VOC, metabolome profiling, combined with a multivariate analysis utilizing total ion currents, was able to distinguish naturally spoiled pork samples from those artificially contaminated with *Salmonella typhimurium*, a food poisoning pathogen commonly recovered from pork products [62]. Levels of a total of 16 compounds, including phenylethyl alcohol and dimethyl disulfide, differed between the contaminated and non-contaminated pork samples. However, the identified compounds in such approaches to microorganism-associated VOC may depend on the multivariate analysis algorithm or other factors originating from meat samples [63]. Impact of other microorganisms (*Pseudomonas* spp., *Brochothrix thermosphacta*, lactic acid bacteria, *Enterobacteriaceae*, and yeasts/molds) on minced beef under the various temperature and packaging conditions was also investigated by a LC–MS approach [64].

With high sensitivity and good selectivity, HS/SPME GC–MS was applied in a study to assess spoilage of minced beef [65]. In this study, the authors focused on influences of temperature and atmosphere in packaging during storage of minced beef with indigenous microorganisms. According to the result, identified compounds such as 2-pentanone and 2-heptanone significantly contributed to unacceptable sensory scores and were associated with spoiled samples. This study showed that the GC–MS-based approach has a potential to estimate microbial counts of the different microorganisms and sensory scores of a meat samples independently of storage conditions.

### 4.7. Authentication

To address the growing consumer awareness, the meat industry has a need for systems to guarantee the authenticity of the meat and the products in order to take measures against increasing fraud/adulteration in the growing complexity of global food chains. Regarding beef, concerns about bovine spongiform encephalopathy (BSE) have increased the awareness of safety. In this field, numerous DNA and protein-based target detection techniques, including species-specific PCR, has been proved to be effective. MEATabolomics is expected to provide useful chemical information regarding authenticity as an alternative method. In this context, analysis of geographic origin of beef by metabolomics was attempted with NMR [66] and imaging MS (IMS) [67]. In the NMR approach, extracts of imported beef from 4 countries were discriminated in the results of PCA and OPLS–DA. The major compounds responsible for the discrimination contained succinate and some of amino acids, suggesting that these compounds could be potential biomarkers to discriminate the geographic origin of beef and that NMR has potential to efficiently work for this analysis. IMS-based direct analysis visualizes distribution of target biomolecules such as lipids, glycolipids, and peptides on biological tissue section samples, without complicated pretreatment procedures. In a matrix-associated laser desorption/ionization–IMS analysis of beef from 3 different Japanese regions, the three types of beef were discriminated by subsequent PCA [67]. In these analyses of geographic origin of beef, although geographic difference in metabolome changes may be due to multiple factors of animal breeds, feeds, and environment, including water and climate, the results revealed availability of metabolomics to distinguish production regions. Geographic differences may also contain the production systems of animals. A study aiming to discriminate the pre-slaughter production system was able to distinguish differences between 1-year cattle fed with barley-based concentrate indoors and with pasture feeding outdoors, using NMR metabolomics followed by PLS–DA, analyzing the *longissimus dorsi* (LD) muscle [68]. The results suggested that NMR approach is suitable for authentication of cattle production history.

Challenges to discriminate beef, pork, and mixture samples by metabolomics were also conducted recently for the establishment of measures against meat adulteration. To this end, different grades of minced beef and pork samples were mixed in various ratios and analyzed by GC–MS and reverse-phase LC–MS approaches [69]. The metabolite content and percentages of fat declared on meat product labeling were correlated each other, and the species of meat was identified by chemometrics using differential metabolite sets. Another volatilomic approach using HS/SPME GC–MS was also applied for the similar purpose [70]. In this study, multiple volatile compounds correlated not only to beef and pork but also the mixture was identified. With use of datasets divided 70% for model calibration and 30% for model prediction, the overall correct classification rate was 99% on average in both datasets. As the authors concluded, this volatilomic approach could be developed for robust and reliable off-line discrimination of meat samples. LC–MS approaches were also applied to classification of chicken meats into normally slaughtered and dead on arrival [71,72]. Such applications along with the development of discrimination analysis is expected to solve the current fraud issues related not only to chicken but also beef and pork.

On the other hand, the irradiation of meat is currently highlighted as an issue for its negative impact in cases when used out of appropriate range of strength, despite positive evaluation of the irradiation effect on meat such as disinfestation, growth inhibition, parasite control, reduction of pathogenic bacteria, and shelf-life extension. Metabolomics is expected to be a tool to monitor the negative impact of irradiation, but it needs to be a non-time-consuming, non-invasive, and reproducible method. In this regard, NMR metabolomics seems suitable for such purpose, and, accordingly, has been used in attempts to investigate the influence of irradiation on beef [73,74]. In the analysis targeting lipids, stepwise linear discriminant analysis (sLDA) following the NMR data profiling allowed the classification of 81.9% of the beef samples according to the irradiation dose (0, 2.5, 4.5, and 8 kGy) [73]. Moreover, the NMR analysis targeting hydrophilic compounds, combined with subsequent classification tree (CT) analysis, was able to distinguish between the irradiated and non-irradiated beef samples [74]. In addition, glycerol, lactic acid esters, and tyramine were found to be important biomarkers for the classification. Thus, MEATabolomics has been applied in a variety of research fields associated with meat production methods and the quality traits, including authentication.

## 5. Conclusions

MEATabolomics has allowed us to better understand skeletal muscle physiology in animals and molecular factors associated with meat quality. Information raised in MEATabolomics can be used as phenotypic indices of muscle properties and meat quality traits, which connects the external phenotype of meat (the quality traits) to regulatory factors in animals or conditions in the production systems. The techniques for metabolomics have been progressing, especially in the process of statistical data analyses, as shown in some examples of attempts to introduce new algorithms and to develop models for phenotype prediction. On the other hand, metabolomics targeting animal blood and meat exudate metabolome, along with use of meat metabolome data, would promote the prediction of meat quality as a non-invasive methodology by utilizing these data. Further studies on the associations of muscles and meat with animal development, stress conditions, welfare and sustainability issues, cooking methods, consumer acceptability, and sensory characteristics would be future challenges for MEATabolomics to be applied to, despite the complex biological processes during meat production and difficulties in chemical identification of secondary metabolites observed in processed and cooked meat. Nevertheless, MEATabolomics is expected to further expand comprehensive association studies with genomics or transcriptomics in an integrative fashion, being supported by the development of new detecting techniques such as REIMS and statistical analytic resources.

## Figures and Tables

**Table 1:** Features of the most commonly used separation techniques in mass spectrometry (MS)-based metabolomics.

	CE	GC	LC
Favorable target metabolites	Polar, charged	Mainly volatile	Non-polar, neutral
Sample derivatization	Unnecessary	Required for non-volatile compounds	Unnecessary
Number of theoretical plate	10^5^ ~ 10^6^	10^4^ ~ 10^5^	10^4^
Separation of structural isomer	High	High	Low
Running time	>20 min	20–40 min	15–40 min
Downstream ionization	ESI	EI, CI	ESI, APCI

**Table 2:** Overview of MEATabolomics studies cited in this review.

Category of Objective	Species/Meat Type	Factors Analyzed	Methodology	Multivariate Data Analysis	Ref.	Authors
Meat characterization	Cattle	Muscle type	HR–MAS ^1^H–NMR	PLS–DA, OPLS–DA	[39]	Ritota et al.
	Lamb	Storage time, display time, packaging conditions	HILIC–MS	PCA	[40]	Subbaraj et al.
	Chicken	pHu	^1^H–NMR	OPLS–DA, MSEA	[41]	Beauclercq et al.
	Beef	Flavor	GC–MS	-	[42]	Takakura et al.
	Beef	Flavor, aging period	HS/SPME GC–MS	-	[43]	Watanabe et al.
	Beef, pork, chicken	Flavor, species, breeds, tissues	GC–MS	OPLS–DA	[44]	Ueda et al.
	Chicken	Age of chicken, muscle type	^1^H–NMR	PLS–DA	[45]	Xiao et al.
Meat abnormality	Chicken	Dystrophy of breast	HR–MAS ^1^H–NMR	PCA, OPLS–DA	[46]	Sundekilde et al.
	Chicken	Wooden breast	GC–MS, LC–MS/MS	RF	[47]	Abasht et al.
	Chicken	Wooden breast	^1^H–NMR	OPLS–DA	[48]	Wang et al.
	Chicken	Wooden breast	^1^H–NMR	OPLS–DA	[49]	Xing et al.
	Chicken	White striping	GC–MS, LC–MS	PCA, Pathway	[50]	Boerboom et al.
Genetic background	Pig	Crossbreeds	^1^H–NMR	PLS	[6]	Straadt et al.
	Pig	Drip loss, association with SNP	GC–MS, LC–MS	Pathway, GWAS	[35]	Welzenbach et al.
	Cattle	Genetic parameters for growth and precocity	^1^H–NMR	PLS–DA	[51]	Consolo et al.
	Cattle	Genetic parameters for chemical traits	GC, LC	-	[52]	Sakuma et al.
	Cattle	NT5E genotype	GC, LC	-	[53]	Komatsu et al.
Animal feeding	Cattle	Grass-fed/grain-fed	GC–MS, LC–MS/MS	PCA, RF	[20]	Carrillo et al.
	Cattle	Dietary amino acid supplementation	^1^H–NMR	PCA	[21]	Yu et al.
	Cattle	Dietary mate extract supplementation	^1^H–NMR	PCA	[22]	de Zawadzki et al.
	Pig	Clenbuterol supplementation	GC–MS	PCA, PLS–DA, OPLS–DA	[23]	Li et al.
	Chicken	Lysine supplementation	CE–MS	-	[24]	Watanabe et al.
	Chicken	Age	^1^H–NMR	PCA, OPLS–DA	[25]	Liu et al.
	Pig	Ractopamine supplementation	REIMS	PCA, LDA, OPLS–DA	[26]	Guitton et al.
Postmortem aging	Pork	Pm. aging period, muscle type	CE–MS	PCA	[14]	Muroya et al.
	Beef	Pm. aging period, muscle type	LC–MS	PCA	[15]	Ma et al.
	Pork	Pm. aging period, muscle type	UPLC–MS/MS	PCA	[16]	Yu et al.
	Beef	Pm. aging period	CE–MS	PCA	[17]	Muroya et al.
	Beef	Pm. aging period	^1^H–NMR	OPLS–DA	[18]	Kodani et al.
	Beef	Pm. aging period	^1^H–NMR	PCA	[19]	Graham et al.
	Pork	Pm. aging period, muscle type (on thiamine)	CE–MS	-	[54]	Muroya et al.
	Beef	Pm. aging period	LC–MS	PCA	[55]	Lana et al.
	Beef	Pm. period of dry-aging	^1^H–NMR	-	[56]	Kim et al.
	Lamb	Fast chilling effect	LC–MS, ^1^H– and ^31^P–NMR	PCA	[57]	Warner et al.
	Beef	Pm. aging period (on oxidative stability)	GC–MS	PCA	[58]	Mitacek et al.
Processing	Pork	Marination time	^1^H–NMR	PCA, OPLS–DA	[30]	Yang et al.
	Pork	Drying/aging period, fermentation of sausage	HR–MAS ^1^H–NMR	PCA	[59]	García-García et al.
Processing, authentication	Pork	Geographic origin, processing method	CE–MS	PCA	[27]	Sugimoto et al.
	Pork	Geographic origin, processing method	^1^H–NMR	PCA, OPLS–DA	[28]	Zhang et al.
Processing, Spoilage	Chicken	Marinade type, storage time, microbial load, sensory score	GC–MS	PCA, FDA	[7]	Lytou et al.
	Chicken	Marinade type, marination time and temperature	LC	PCA	[29]	Lytou et al.
Sensory evaluation	Beef	Grinding score, packaging method	LC–MS	PCA, PLS	[60]	Jiang et al.
	Beef	Commercial brands	GC–MS	–	[61]	Suzuki et al.
Spoilage	Pork	Salmonellae contamination, time of microbial exposure	GC–MS	PCA, etc.	[62,63]	Xu et al.
	Beef	Packaging, temperature	LC–MS	PCA, FDA, PLS–R	[64]	Argyri et al.
	Beef	Packaging, temperature, sensory score, microbial growth	HS/SPME GC–MS	PCA, FDA, PLS–R	[65]	Argyri et al.
Authentication	Beef	Geographic origin	^1^H–NMR	PCA, OPLS–DA	[66]	Jung et al.
	Beef	Geographic origin	IMS	PCA	[67]	Zaima et al.
	Beef	Production system	^1^H–NMR	PLS–DA	[68]	Osorio et al.
	Beef, Pork	Species	GC–MS, UPLC–MS	PCA, PLS–DA, Pathway	[69]	Trivedi, et al.
	Beef, Pork	Species	HS/SPME GC–MS	PCA, PLS–DA	[70]	Pavlidis et al.
	Chicken	Live/dead on arrival	LC–MS	PCA	[71]	Sidwick et al.
	Chicken	Live/dead on arrival	LC–MS	PCA, Pathway	[72]	Cao et al.
	Beef	Irradiation doses (on lipids)	^1^H–NMR	sLDA, ANN	[73]	Zanardi et al.
	Beef	Irradiation doses (on hydrophilic compounds)	^1^H–NMR	PCA, CT	[74]	Zanardi et al.

ANN: artificial neural networks; CT: classification tree; FDA: factorial discriminant analysis; GWAS: genome-wide association analysis; HILIC: hydrophilic interaction liquid chromatography; HS/SPME: head space–solid phase microextraction; LDA: linear discriminant analysis; MSEA: metabolite set enrichment analysis; OPLS–DA: orthogonal PLS–discrimination analysis; Pathway: pathway enrichment analysis; Pm.: postmoretm; PCA: principal component analysis; PLS: partial least square analysis; PLS–DA: PLS–discrimination analysis; REIMS: rapid Evaporative Ionization Mass Spectrometry; RF: random forest; sLDA: stepwise linear discriminant analysis; UPLC: ultra-performance LC.
